# An Actinide Zintl Cluster: A Tris(triamidouranium)μ_3_-η^2^:η^2^:η^2^-Heptaphosphanortricyclane and Its Diverse Synthetic Utility**

**DOI:** 10.1002/anie.201306492

**Published:** 2013-10-14

**Authors:** Dipti Patel, Floriana Tuna, Eric J L McInnes, William Lewis, Alexander J Blake, Stephen T Liddle

**Affiliations:** School of Chemistry, University of Nottingham University Park, Nottingham, NG7 2RD (United Kingdom) E-mail: stephen.liddle@nottingham.ac.uk; School of Chemistry and Photon Science Institute, University of Manchester Oxford Road, Manchester, M13 9PL (United Kingdom)

**Keywords:** actinides, reduction, uranium, white phosphorus, Zintl phases

Zintl clusters,[1] exemplified by the heptaphosphanortricyclane trianion [P_7_]^3−^, are fundamentally interesting and important structural units in solid-state and molecular chemistry.[1, 2] Their importance derives from the key role they have played in the development of polyhedral bonding models and isoelectronic relationships to cycloalkanes as well as synthetic applications.[3]–[5]

There is currently major interest in the activation of elemental phosphorus.[6] This is because phosphorus-containing molecules are ubiquitous and form the basis of numerous industries, yet their synthesis relies on chlorination of P_4_ to give PCl_3_ followed by multistep derivatizations. A highly attractive concept is to avoid the need for PCl_3_ and access organophosphorus compounds directly from elemental phosphorus. In principle, [P_7_]^3−^ is an attractive precursor to organophosphorus derivatives; however, although Group 1 derivatives can be prepared straightforwardly in liquid ammonia, high-temperature melts have a reputation for detonating in the presence of traces of moisture, and Na/K reduction of phosphorus in ethers gives non-stoichiometric mixtures.[7] Unlike main-group and late-transition-metal-mediated activation of P_4_,[8, 9] examples of early metal-mediated transformations of P_4_ are far less common.[10] In Group 3 and 4f-block chemistry, despite the potentially strongly reducing nature of these metals, activation of P_4_ is surprisingly rare,[11] presumably because of the hard–soft mismatch between the electropositive metal and soft phosphorus.[12]

For 5f metals, reports of P_4_ activation are exceptionally rare; there is one report of thorium-mediated activation of P_4_ at elevated temperature or with co-reagents,[13] and only two examples of uranium-mediated activation of P_4_ are known.[14] However, for both uranium cases it is notable that no fragmentation or catenation of P_4_ was observed and instead only cleavage of two of the P–P bonds in P_4_ to give [P_4_]^2−^ rings was observed. Indeed, early metal-mediated conversion of P_4_ to [P_7_]^3−^ is in general a rare occurrence.[6, 11c,d] Herein, we report that a diuranium(V)–arene-tetraanion complex reductively cleaves P_4_ to selectively form a triuranium heptaphosphanortricyclane cluster under mild conditions. This cluster is the first example of a molecular actinide [P_7_] Zintl complex and the first example of fragmentation and catenation of P_4_ to a higher oligomer promoted by uranium. Additionally, it is notable that no binary uranium phosphides are formed. Furthermore, we show that this complex is a precursor to a wide range of facile derivatization reactions in closed synthetic cycles for the activation and functionalization of P_4_ under mild conditions.

Treatment of [{U(Ts^Tol^)}_2_(μ-η^6^:η^6^-C_6_H_5_CH_3_)][15] (**1**, Ts^Tol^=HC(SiMe_2_NAr)_3_; Ar=4-MeC_6_H_4_) with P_4_ (1:1.1 of **1**:P_4_) afforded, after work-up and isolation, brown crystals of the Zintl complex [{U(Ts^Tol^)}_3_(μ_3_-η^2^:η^2^:η^2^-P_7_)] (**2**) in 12 % yield of crystalline product (Scheme 1). This low yield reflects the surprisingly high solubility of **2**, but by ^1^H NMR spectroscopy we estimate about 65 % of the crude reaction mixture is **2**, with some protonated ligand present, presumably from minor decomposition.[16] The ^1^H NMR spectrum of crystalline **2** is broad and the complex is silent in the ^31^P NMR spectrum in the range ±1000 ppm, which is most likely as a result of a combination of a reduction of the intensities of resonances owing to extensive ^*n*^*J*_PP_ couplings (*n*=1, 2, 3),[2]–[5, 17] and line-broadening that is due to dynamic processes and fast relaxation from the presence of coordinated uranium centers. Variable-temperature ^1^H and ^31^P NMR spectroscopy could not freeze out any dynamic processes or induce coalescence to one time-averaged species, which is most likely due to the effects described above. Germane to this point, the [P_7_]^3−^ trianion is well-known to undergo facile and very complicated Cope-type rearrangements in solution that are similar to bullvalene,[18] and we suggest this contributes to the origin of the broad NMR resonances. The magnetic moment of pure **2** in solution was found to be 4.67 μ_B_ at 298 K. In reasonable agreement with this, the magnetic moment of powdered **2** was found to be 4.20 μ_B_ at 298 K; this decreases slowly on cooling down to ca. 80 K before falling more precipitously, reaching 1.25 μ_B_ at 1.8 K and still decreasing. The room-temperature moment corresponds to 2.42 μ_B_ per uranium(IV) ion; this is significantly lower than the value calculated for a free ^3^H_4_ term (3.58 μ_B_), but this is a common observation for uranium(IV). For example, [U(Ts^Xy^)Co(CO)_3_(PPh_3_)] (Xy=xylyl; the Co is diamagnetic) contains a single uranium(IV) ion with a near identical capping ligand and has a room-temperature moment of 2.77 μ_B_,[19] close to the value per uranium ion we observe for **2** and this supports a uranium(IV) formulation. It is noticeable that the magnetic moment of **2** decreases with decreasing temperature more slowly than is commonly observed for uranium(IV), but this has been observed before in [U(Ts^Xy^)Co(CO)_3_(PPh_3_)] and [UO(N′′)_3_][CoCp*_2_] (N′′=N(SiMe_3_)_2_), which both contain uranium(IV) centers.[19, 20] The electronic absorption spectrum of **2** is dominated by charge transfer bands in the 25 000–12 000 cm^−1^ region, and a number of surprisingly intense absorptions (*ε*=120–250 L mol^−1^ cm^−1^) that are as assigned as f→f transitions are observed in the 12 000–5000 cm^−1^ region; the presence of the latter supports the uranium(IV) formulation.

**Scheme 1:**
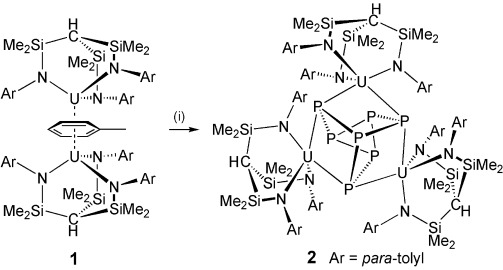
Synthesis of 2. Reagents and conditions: i) P_4_ (1.1 equiv) in toluene, −toluene. Ar=*p*-tolyl.

To confirm the structure of **2**, the X-ray crystal structure was determined (Figure 1).[21] The salient feature of **2** is the formation of a [P_7_]^3−^ trianion that bridges three [U(Ts^Tol^)]^+^ fragments, where each uranium center coordinates to two phosphorus centers on the upper rim of the [P_7_]^3−^ trianion. The U–P bond lengths span the range 2.9486(17)–3.0308(17) Å, which compares to the sum of the covalent radii of 2.81 Å for uranium and phosphorus,[22] and most likely reflects the sterically demanding nature of the {U(Ts^Tol^)}^+^ fragments and the bridging coordination mode. The U–N and P–P bond lengths are unexceptional. A common parameter used to assess the extent of ionic character in [P_7_]^3−^ trianions is the Q value,[1, 7e, 23] where *Q*=*h*/*a* (*h*=distance from the apical P-center to the center-point of the lower rim of three P-centers; *a*=average P–P distance in the lower rim). For ionic systems, the *Q* value is typically 1.3–1.4 (for example, in P_7_(SiR_3_)_3_ derivatives), and for **2** the *Q* value is 1.39, which is suggestive of predominantly electrostatic U–P bonding. The computational size of **2** rendered a full DFT analysis of **2** intractable, but a preliminary single-point energy calculation on **2** revealed the U–P interactions to be essentially ionic, which is in agreement with the structural and NMR spectroscopic data.

**Figure 1:**
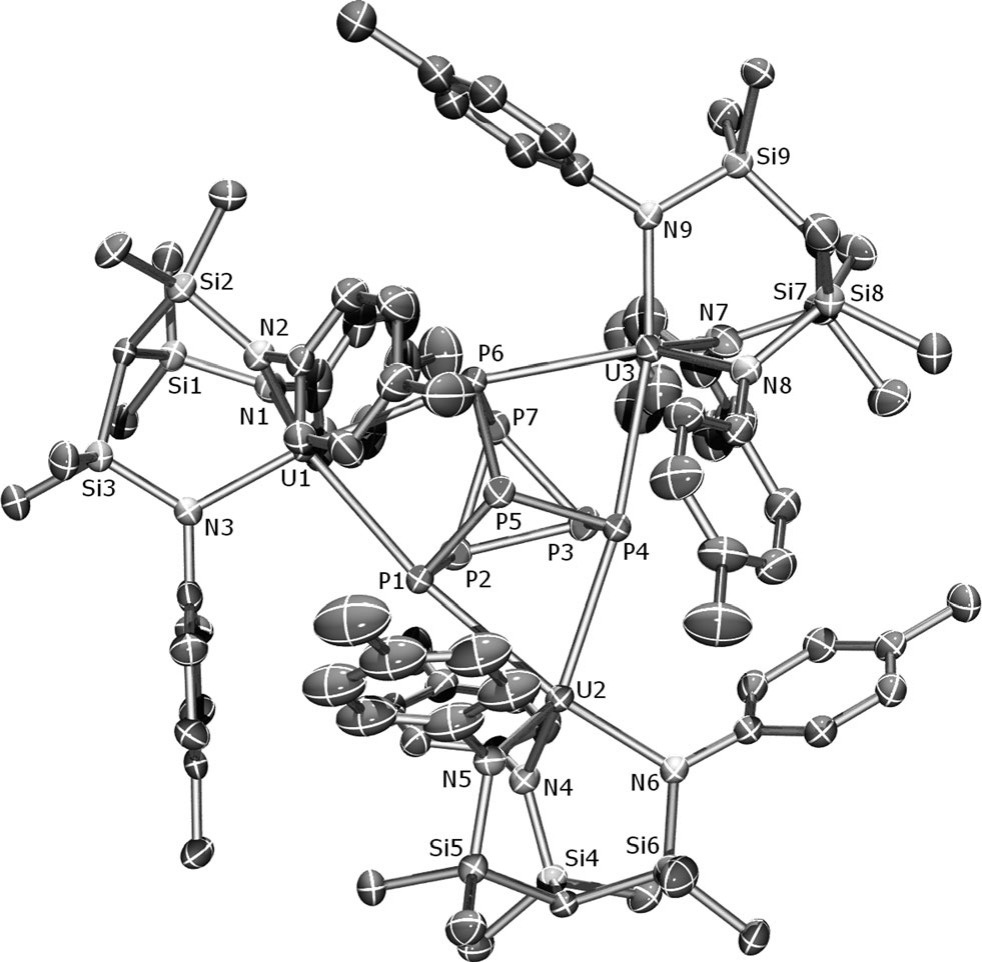
Molecular structure of 2 with ellipsoids set at 40 % probability; hydrogen atoms, minor disorder components, and lattice solvent are omitted for clarity.

With **2** in hand, we undertook preliminary experiments to explore its synthetic utility under ambient conditions, Scheme 2,[16] because, although the activation of white phosphorus is burgeoning, subsequent functionalization and liberation reactions are not common[6, 24] and are often limited to silyl derivatives.[11c] To benchmark the reactivity of **2**, we treated **2** with three equivalents of Me_3_SiCl to quantitatively afford P_7_(SiMe_3_)_3_[25] and [(Ts^Tol^)U(Cl)(μ-Cl)U(THF)_2_(Ts^Tol^)][26] (**3**, after the addition of THF); both of these compounds are known and were identified by ^1^H and ^31^P NMR spectroscopies. Encouraged by the facile reactivity of **2**, we examined more challenging electrophiles. Lithium chloride reacts quantitatively with **2** to afford **3** (after the addition of THF and tmeda) and P_7_[Li(tmeda)]_3_.[27] This is notable because alkali-metal derivatives of [P_7_]^3−^ can be difficult to prepare. In the context of organophosphorus derivative chemistry, we find that excess methyl iodide and phenyl iodide both react cleanly and quantitatively with **2** to afford P_7_(Me)_3_[28] and P_7_(Ph)_3_,[29] respectively, with concomitant formation of **3** (after the addition of THF), whereas these phosphanortricyclanes were previously not straightforward to prepare. This broad palate of reactions establishes that sp^3^ and sp^2^ (aromatic) carbon-based electrophiles can be substituted onto the [P_7_]^3−^ framework in P–C bond forming reactions from **2**, thus providing extensive opportunities for subsequent functionalization and derivatization chemistry.

**Scheme 2:**
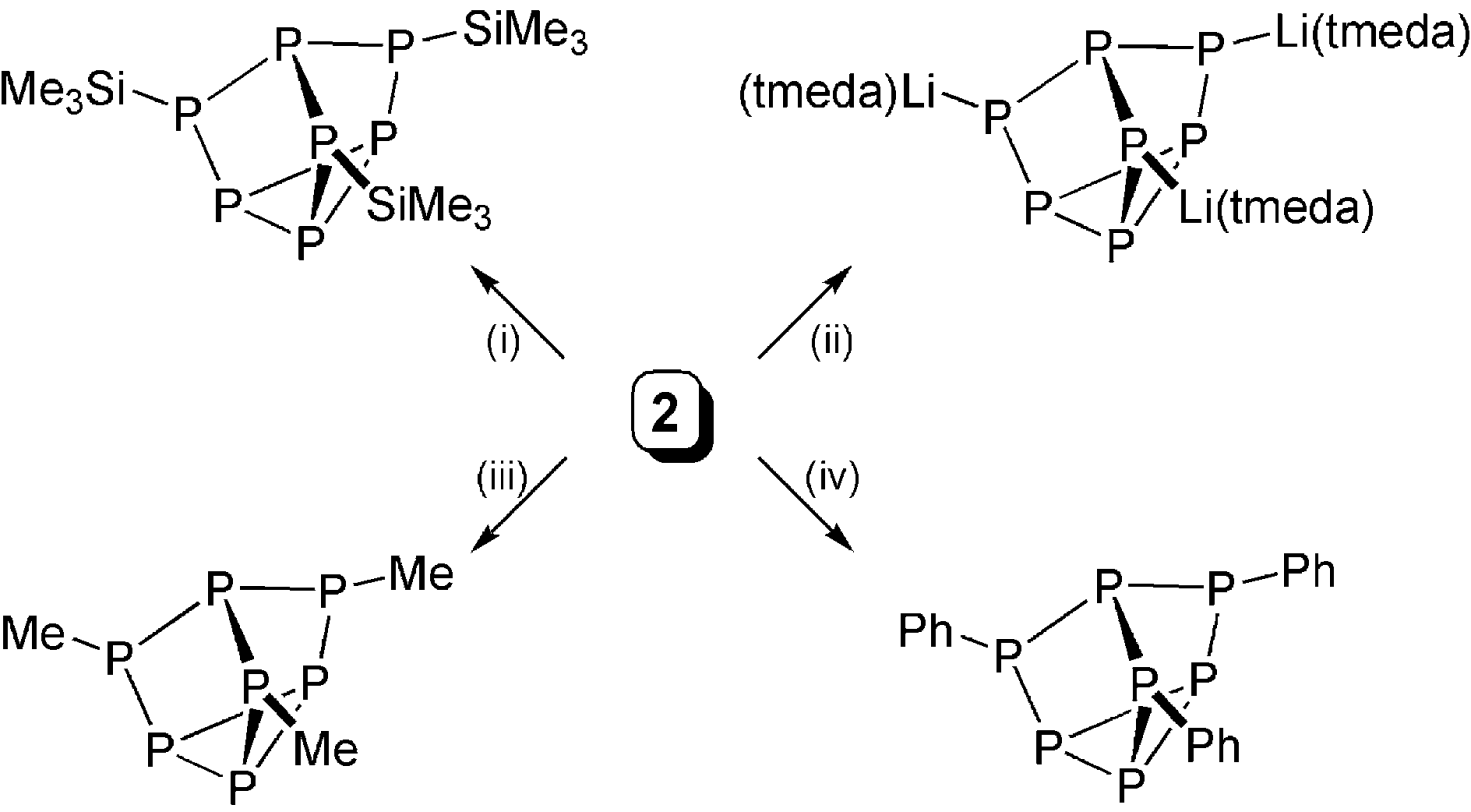
Reactions of 2 with electrophiles. Reagents and conditions (all at RT): i) excess Me_3_SiCl, C_6_D_6_, THF, −3; ii) 3 LiCl, C_6_D_6_, THF, 3 tmeda, −3; iii) excess MeI, C_6_D_6_, THF, −3; iv) PhI, C_6_D_6_, THF, −3.

As **3** is the direct precursor to **1**, the derivatization chemistry described herein presents the closure of synthetic cycles for the activation and functionalization of white phosphorus (Scheme 3). In practice, two turnovers could be achieved before the mixture of products rendered subsequent reactions unfeasible. The yields of the polyphosphide derivatives for the first turnover were generally quantitative, but this dropped to circa 40 % in the second turnover, reflecting the buildup of inorganic salts and the diminishing yields of **1** and **2** in each turnover. However, the diverse and straightforward nature of these reactions suggests that **2** is amenable to reactions with a wide range of functional electrophiles, and reactions to extend the scope and efficacy of this reactivity are ongoing.

**Scheme 3:**
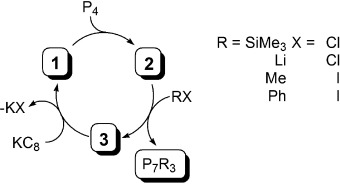
Synthetic cycle for the catenation and functionalization of white phosphorus by 1 via 2.

To conclude, the reaction of P_4_ with [{U(Ts^Tol^)}_2_(μ-η^6^:η^6^-C_6_H_5_CH_3_)] affords the first example of an actinide [P_7_] Zintl complex and the first example of fragmentation and catenation of P_4_ promoted by uranium. This Zintl complex is a precursor to a range of derivatives that represent general methods for the preparation of alkali-metal-, hydrocarbon-, aromatic-, and silyl-functionalized P_7_ derivatives via Li–P, P–C (sp^2^- and sp^3^-hybridized carbon groups), and P–Si bond formation reactions. This offers significant synthetic scope for the closure of synthetic cycles for the activation and functionalization of P_4_ under mild conditions and further demonstrates the ability of triamido uranium complexes to activate and liberate functionalized small molecules.[30]

## Experimental Section

Synthesis of **2**: Toluene (20 mL) was added to a cold (−78 °C) stirring mixture of **1** (0.79 g, 0.50 mmol) and P_4_ (0.07 g, 0.55 mmol). The mixture was stirred at −78 °C for 5 min, then was allowed to warm to room temperature, and it was then stirred for a further 16 h. All volatiles were removed in vacuo. The product was extracted into hexanes (10 mL), filtered, and the hexanes extract was stored at room temperature for 16 h to yield large brown blocks of **1** (Crystalline yield: 0.10 g, 12 %). Inspection of the crude reaction mixture showed that **2** constitutes about 65 % of the reaction mixture. Anal. calcd. (%) for C_84_H_120_N_9_P_7_Si_9_U_3_⋅C_6_H_14_: C 42.80, H 5.35, N 4.99 %; found: C 42.63, H 5.34, N 4.82 %. ^1^H NMR (C_6_D_6_): *δ*_H_=15–11.50 (27 H, vb s, Ar-C*H*_3_), 1.75–0 (36 H, vb s, o and *m*-C*H*), −1.75–−4.50 (54 H, v br s, SiC*H*_3_), −73.31 ppm (3 H, s, Si–C*H*). Magnetic moment (Evans’ method, C_6_D_6_, 298 K): *μ*_eff_=4.67 μ_B_. FTIR (Nujol): 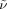
1604 (w), 1513 (w), 1495 (s), 1403 (w), 1364 (w), 1286 (w), 1251 (m), 1243 (m), 1221 (s), 1171 (w), 1102 (w), 1015 (w), 974 (m), 933 (m), 899 (s), 858 (s), 841 (vs), 810 (s), 765 (w), 708 (m), 697 (w), 543 (w), 503 (m) cm^−1^. UV/Vis/NIR (toluene): *λ*_max_ (ε/L mol^−1^ cm^−1^): 1033–1139 (217, 200), 1457 (119), 1622 (130), 1844 (151), 2053–2278 (158, 146).

## Supporting Information

As a service to our authors and readers, this journal provides supporting information supplied by the authors. Such materials are peer reviewed and may be re-organized for online delivery, but are not copy-edited or typeset. Technical support issues arising from supporting information (other than missing files) should be addressed to the authors.

miscellaneous_information

## References

[1] Scharfe S, Kraus F, Stegmaier S, Schier A, Faessler TF (2011). Angew. Chem.

[b01] (2011). Angew. Chem. Int. Ed.

[2] Baudler M, Glinka K (1993). Chem. Rev.

[3] Baudler M (1982). Angew. Chem.

[b02] (1982). Angew. Chem. Int. Ed. Engl.

[4] Baudler M, Glinka K (1994). Chem. Rev.

[5] Kraus F, Korber N (2005). Chem. Eur. J.

[6] Cossairt BM, Piro NA, Cummins CC (2010). Chem. Rev.

[7a] Santandrea RP, Mensing C, von Schnering HG (1986). Thermochim. Acta.

[7b] Korber N, Daniels J (1996). Helv. Chim. Acta.

[7c] Korber N, von Schnering HG (1996). Chem. Ber.

[7d] Korber N, Daniels J (1996). J. Chem. Soc. Dalton Trans.

[7e] Noblet P, Cappello V, Tekautz G, Baumgartner J, Hassler K (2011). Eur. J. Inorg. Chem.

[8] Scheer M, Balázs G, Seitz A (2010). Chem. Rev.

[9] Caporali M, Gonsalvi L, Rossin A, Peruzzini M (2010). Chem. Rev.

[10a] Hey E, Lappert MF, Atwood JL, Bott SG (1987). J. Chem. Soc. Chem. Commun.

[10b] Scherer OJ, Swarowsky M, Swarowsky H, Wolmershäuser G (1988). Angew. Chem.

[b03] (1988). Angew. Chem. Int. Ed. Engl.

[10c] Chirik PJ, Pool JA, Lobkovsky E (2002). Angew. Chem.

[b04] (2002). Angew. Chem. Int. Ed.

[10d] Seidel WW, Summerscales OT, Patrick BO, Fryzuk MD (2009). Angew. Chem.

[b05] (2009). Angew. Chem. Int. Ed.

[11a] Konchenko SN, Pushkarevsky NA, Gamer MT, Köppe R, Schnöckel H, Roesky PW (2009). J. Am. Chem. Soc.

[11b] Li T, Wiecko J, Pushkarevsky NA, Gamer MT, Köppe R, Konchenko SN, Scheer M, Roesky PW (2011). Angew. Chem.

[b06] (2011). Angew. Chem. Int. Ed.

[11c] Huang W, Diaconescu PL (2012). Chem. Commun.

[11d] Huang W, Diaconescu PL (2013). Eur. J. Inorg. Chem.

[12a] Clegg W, Izod K, Liddle ST, O’Shaughnessy P, Sheffield JM (2000). Organometallics.

[12b] Izod K, O’Shaughnessy P, Sheffield JM, Clegg W, Liddle ST (2000). Inorg. Chem.

[12c] Izod K, Liddle ST, McFarlane W, Clegg W (2004). Organometallics.

[12d] Izod K, Liddle ST, Clegg W (2004). Chem. Commun.

[12e] Masuda JD, Jantunen KC, Ozerov OV, Noonan KJT, Gates DP, Scott BL, Kiplinger JL (2008). J. Am. Chem. Soc.

[12f] Cui P, Chen Y, Xu X, Sun J (2008). Chem. Commun.

[12g] Lv Y, Xu X, Chem Y, Leng X, Borzov MV (2011). Angew. Chem.

[b07] (2011). Angew. Chem. Int. Ed.

[13] Scherer OJ, Werner B, Heckmann G, Wolmershauser G (1991). Angew. Chem.

[b08] (1991). Angew. Chem. Int. Ed. Engl.

[14b] Frey ASP, Cloke FGN, Hitchcock PB, Green JC (2011). New J. Chem.

[15] Patel D, Tuna F, McInnes EJL, McMaster J, Lewis W, Blake AJ, Liddle ST (2013). Dalton Trans.

[17a] Mills DP, Cooper OJ, Tuna F, McInnes EJL, Davies ES, McMaster J, Moro F, Lewis W, Blake AJ, Liddle ST (2012). J. Am. Chem. Soc.

[17b] King DM, Tuna F, McInnes EJL, McMaster J, Lewis W, Blake AJ, Liddle ST (2013). Nat. Chem.

[17c] Cooper OJ, Mills DP, McMaster J, Tuna F, McInnes EJL, Lewis W, Blake AJ, Liddle ST (2013). Chem. Eur. J.

[18] Baudler M, Ternberger H, Faber W, Hahn J (1979). Z. Naturforsch. B.

[19] Patel D, Moro F, McMaster J, Lewis W, Blake AJ, Liddle ST (2011). Angew. Chem.

[b09] (2011). Angew. Chem. Int. Ed.

[20] Brown JL, Fortier S, Lewis RA, Wu G, Hayton TW (2012). J. Am. Chem. Soc.

[21] http://www.ccdc.cam.ac.uk/data_request/cif.

[22] Pyykkö P, Atsumi M (2009). Chem. Eur. J.

[23] Hönle W, von Schnering HG (1978). Z. Anorg. Allg. Chem.

[24a] Cummins CC (2006). Angew. Chem.

[b010] (2006). Angew. Chem. Int. Ed.

[24b] Piro N, Figueroa JS, McKellar JT, Cummins CC (2006). Science.

[25] Fritz G, Härer J (1983). Z. Anorg. Allg. Chem.

[26] Patel D, Lewis W, Blake AJ, Liddle ST (2010). Dalton Trans.

[27] Hönle W, Von Schnering HG, Schmidpeter A, Burget G (1984). Angew. Chem.

[b011] (1984). Angew. Chem. Int. Ed. Engl.

[28] Baudler M, Faber W, Hahn J (1980). Z. Anorg. Allg. Chem.

[29] Hölderich W, Fritz G (1979). Z. Anorg. Allg. Chem.

[30a] Gardner BM, Stewart JC, Davis AL, McMaster J, Lewis W, Blake AJ, Liddle ST (2012). Proc. Natl. Acad. Sci. USA.

[30b] King DM, Tuna F, McInnes EJL, McMaster J, Lewis W, Blake AJ, Liddle ST (2012). Science.

[30c] Gardner BM, Liddle ST (2013). Eur. J. Inorg. Chem.

