# Supplementary material for: An Actinide Zintl Cluster: A Tris(triamidouranium)μ3-η2:η2:η2-Heptaphosphanortricyclane and Its Diverse Synthetic Utility
Source: Angew Chem Int Ed Engl. 2013 Oct 14;52(50):13334–7. doi: 10.1002/anie.201306492 (PMC4674987; doi:10.1002/anie.201306492)
Supplement: Supplementary file 1 — miscellaneous_information [file anie0052-13334-sd1.pdf]

Angewandte Chemie
Eine Zeitschrift der Gesellschaft Deutscher Chemiker

Supporting Information

© Wiley-VCH 2013

69451 Weinheim, Germany

An Actinide Zintl Cluster: A Tris(triamidouranium)μ_3-η^2:η^2:η^2-Heptaphosphanortricyclane and Its Diverse Synthetic Utility**

*Dipti Patel, Floriana Tuna, Eric J. L. McInnes, William Lewis, Alexander J. Blake, and Stephen T. Liddle**

anie_201306492_sm_miscellaneous_information.pdf

Experimental

General

All manipulations were carried out using Schlenk techniques, or an MBraun UniLab glovebox, under an atmosphere of dry nitrogen. Solvents were dried by passage through activated alumina towers and degassed before use. All solvents were stored over potassium mirrors. Deuterated solvent was distilled from potassium, degassed by three freeze-pump-thaw cycles and stored under nitrogen. [{U(Ts^{Tol})}$_2$(μ-η^6:η^6-$C_6H_5CH_3$)][15] [**1**, Ts^{Tol} = HC($SiMe_2NAr$)$_3$; Ar = 4-Me-C_6H_4] was prepared by the literature methods.[1,2]

^{1}H and ^{31}P NMR spectra were recorded on a Bruker 400 spectrometer operating at 400.2 and 162.0 MHz respectively; chemical shifts are quoted in ppm and are relative to TMS (^{1}H) and external 85% H_3PO_4 (^{31}P). FTIR spectra were recorded on a Bruker Tensor 27 spectrometer. UV/Vis/NIR spectra were recorded on a Perkin Elmer Lambda 750 spectrometer. Data were collected in 1mm path length cuvettes loaded in an MBraun UniLab glovebox and were run versus the appropriate reference solvent. Variable-temperature magnetic moment data were recorded in an applied dc field of 0.1 T on a Quantum Design MPMS XL5 superconducting quantum interference device (SQUID) magnetometer using doubly recrystallised powdered samples. Samples were carefully checked for purity and data reproducibility between several independently prepared batches for each compound examined. Care was taken to ensure complete thermalisation of the sample before each data point was measured and samples were immobilized in an eicosane matrix to prevent sample reorientation during measurements. A diamagnetic correction of 1262.88×10^{-6} cm^3 mol^{-1} was applied for **2** using tabulated Pascal constants and measurements were corrected for the effect of the blank sample holders (flame sealed Wilmad NMR tube and straw) and eicosane matrix. Solution magnetic moments were recorded at room temperature using the Evans method. CHN microanalyses were carried out by Tong Liu at the University of Nottingham.

Derivatisation Reactions of 2

Reactions were conducted according to the following general and representative procedure. Complex **2** (16.3 mg, 6.7 μmol) was dissolved in C_6D_6 (0.55 ml) in a Youngs tap NMT tube and to this was added the substrate (~4 equivalents for liquids, 3 equivalents for LiCl). The NMR tube was sealed and vigorously shaken then ^{1}H and ^{31}P NMR spectra were obtained. Following confirmation that a reaction had occurred THF (2 drops) was added to enable **3** to form. For the LiCl reaction tmeda (3 equivalents) was added to allow the known P_7[Li(tmeda)]$_3$ compound to form. NMR spectra were then re-run to confirm the formation of **3**. Spectroscopic data were compared to literature reports to corroborate the formation of the phosphanortricyclanes.[3-6]

Representative Recycling following Treatment of 2 with Electrophiles

As described above a solution of **2** in C_6D_6 (0.55 ml) was treated with the relevant substrate. Following confirmation of the formation of **3** and the polyphosphorus species two drops of toluene were added along with one equivalent of KC_8 per uranium ion. The NMR tube was sealed and vigorously shaken and then the ^{1}H NMR spectrum was obtained to confirm the formation of **1** (the chemical shift of the methane C-H hydrogen of the Ts-ligand is diagnostic). P_4 (1.1 equivalents) was then added and the tube resealed and shaken. The electrophile was then introduced, and the NMR tube resealed and shaken, and then NMR spectra were obtained. In practice two cycles could be achieved but further recycling proved unworkable.

Electronic Absorption Spectrum of 2 in Toluene

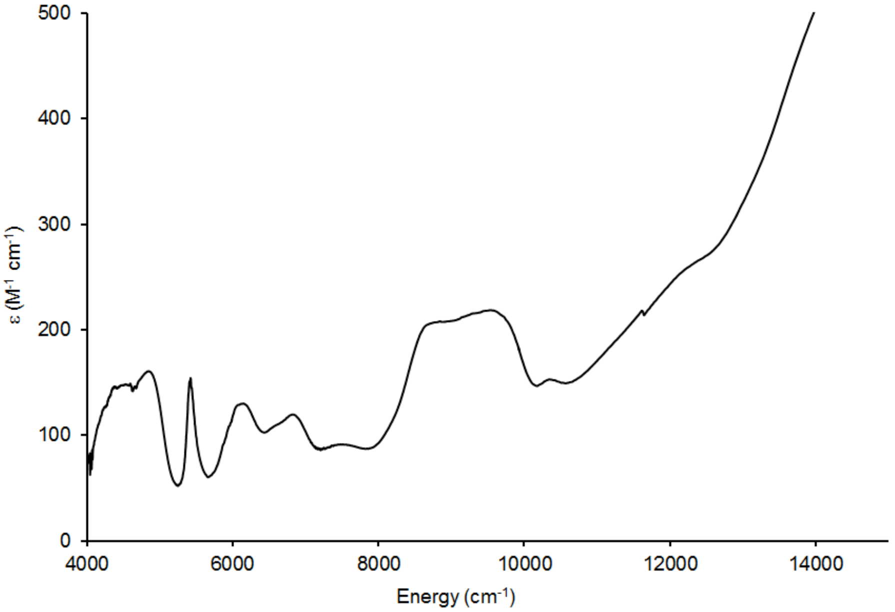

Variable-Temperature SQUID Magnetisation Data for 2

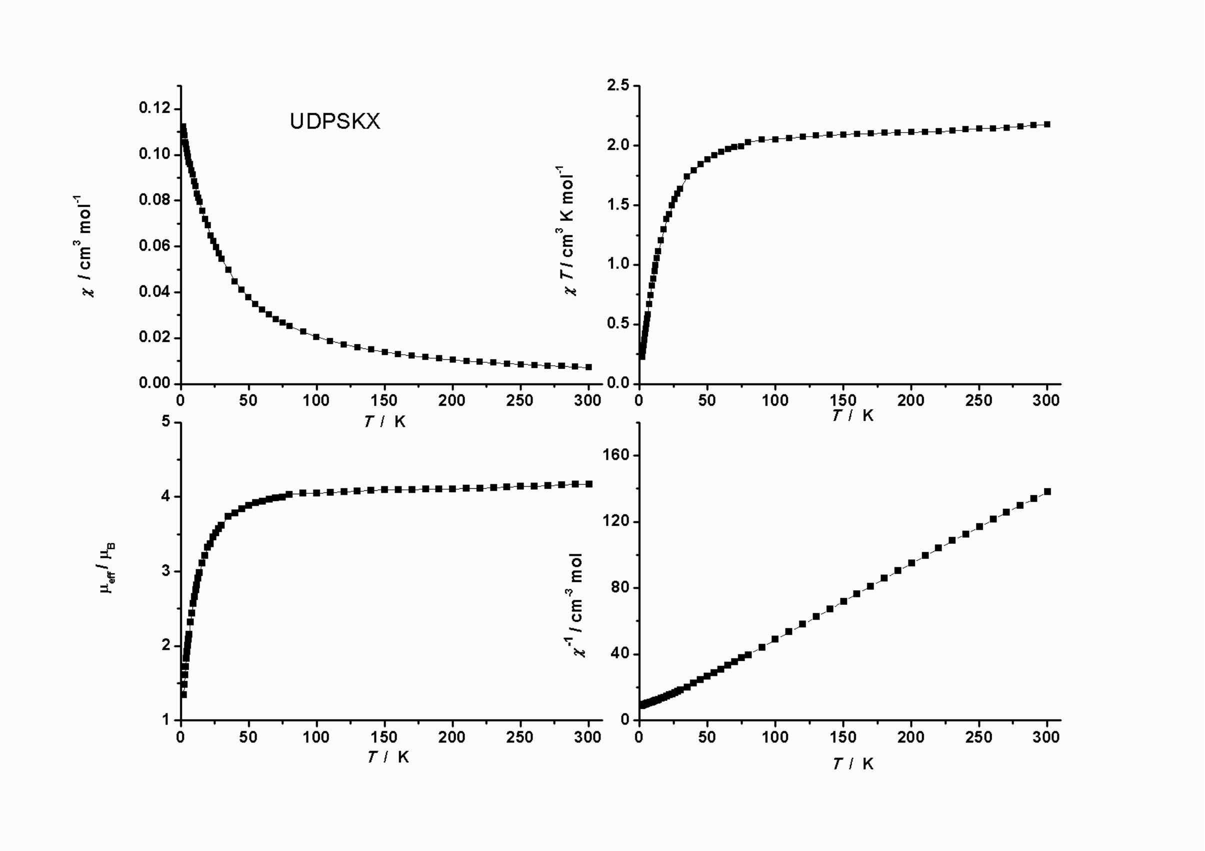

NMR Spectra

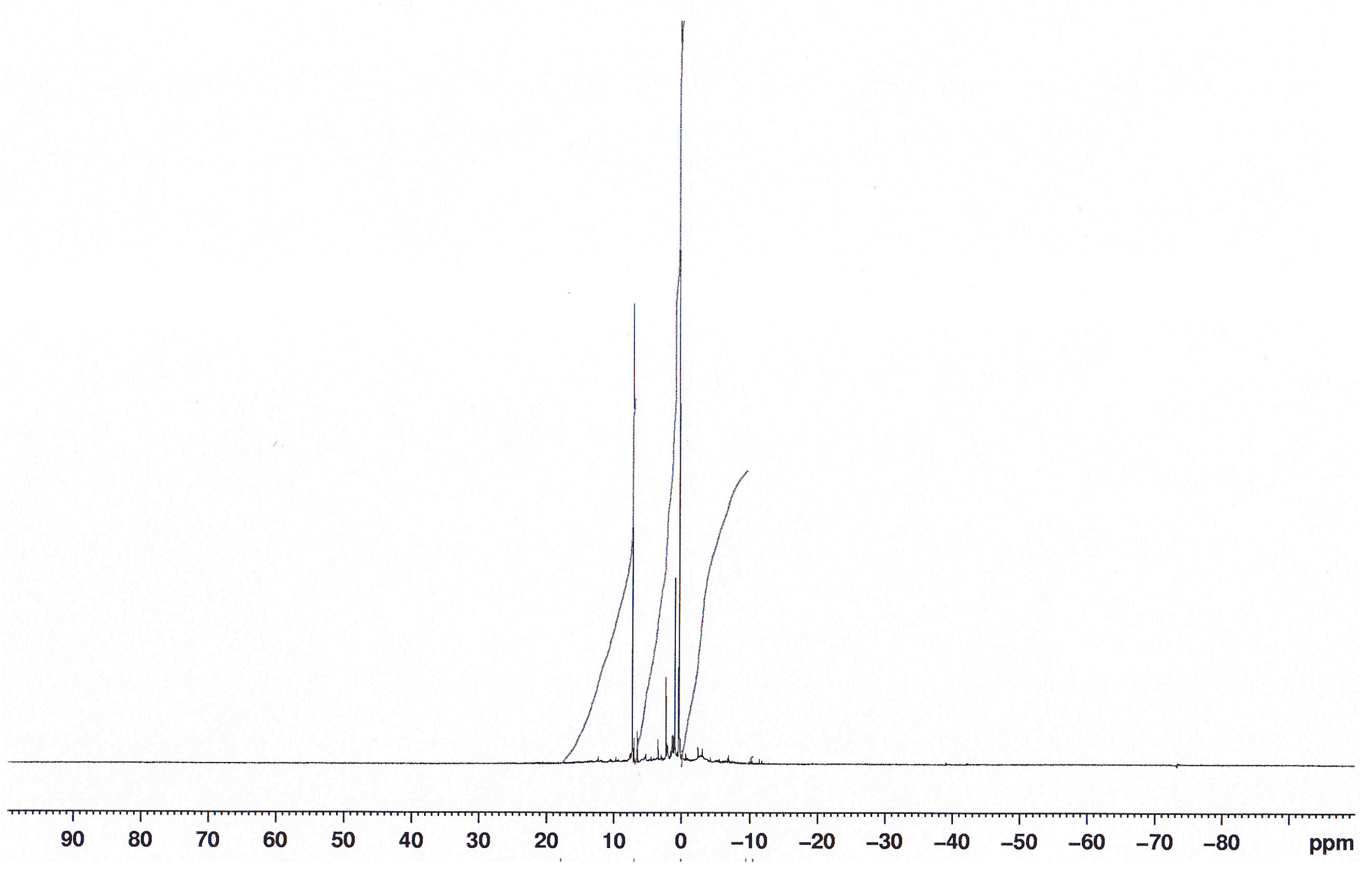

^{1}H NMR Spectrum of Crude Reaction Mixture from Preparation of 2

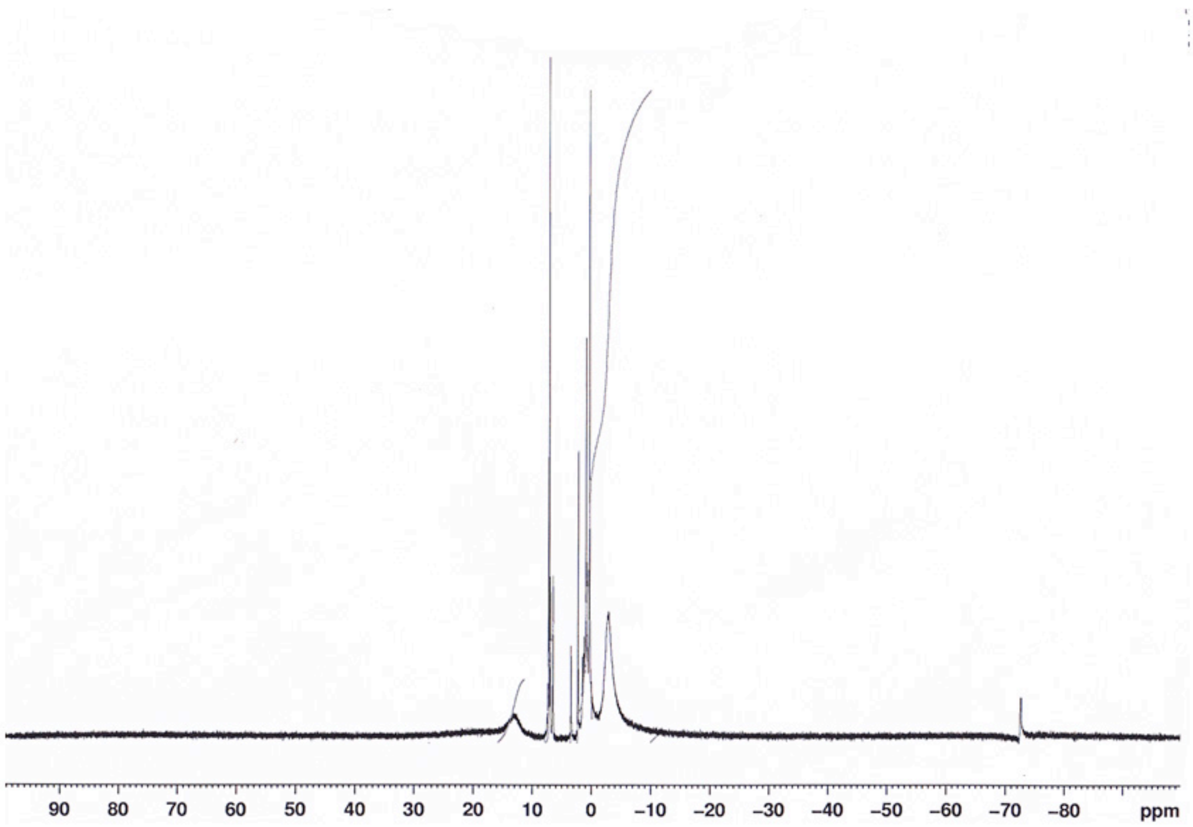

^{1}H NMR Spectrum of Crystalline 2

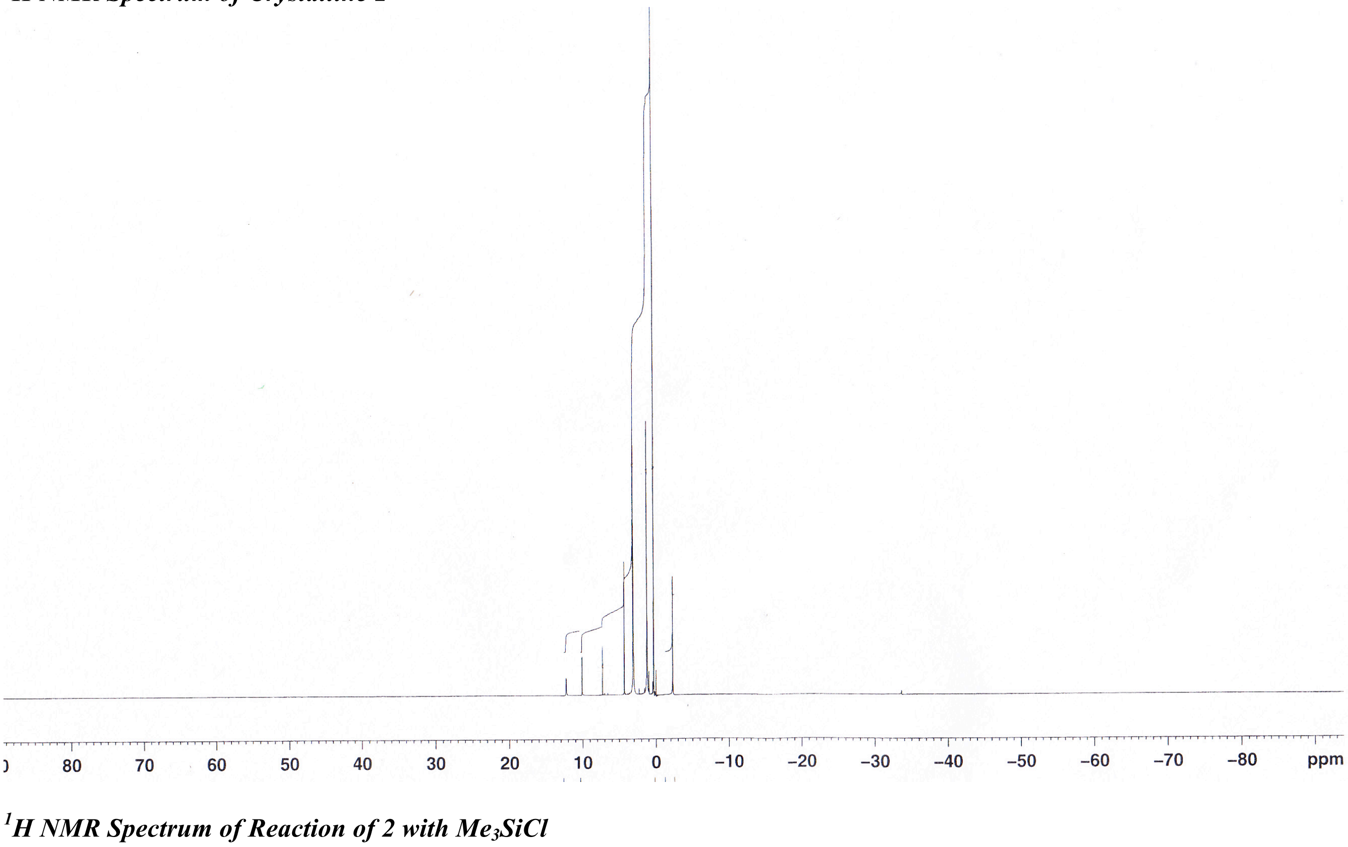

^{1}H NMR Spectrum of Reaction of 2 with Me_3SiCl

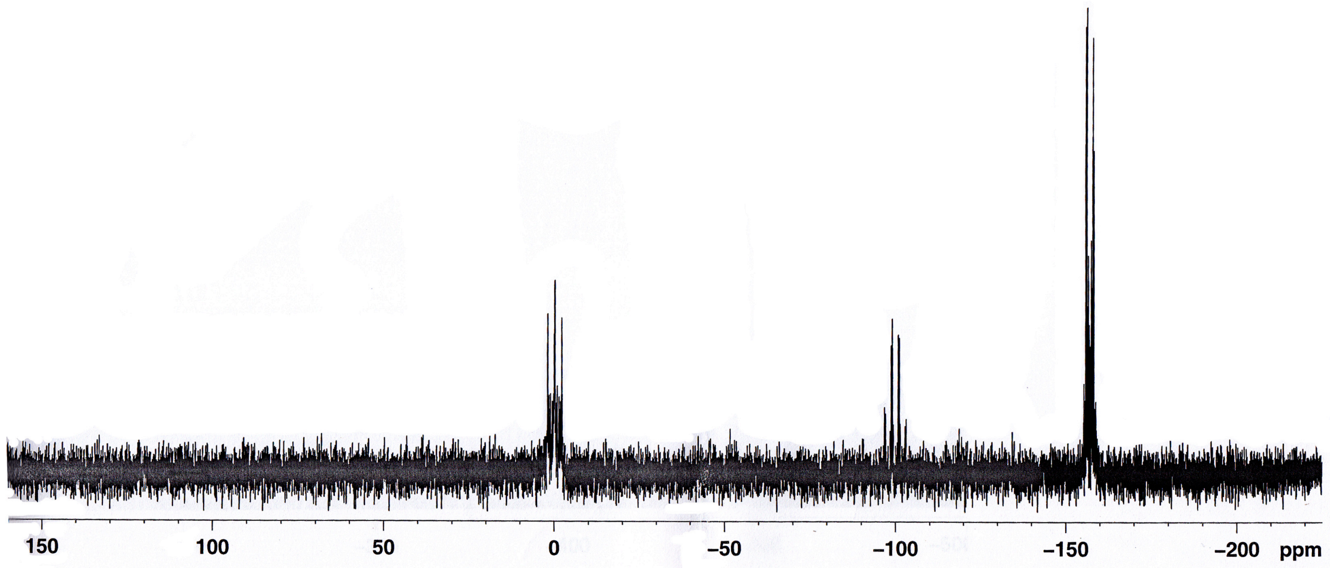

^{31}P NMR Spectrum of Reaction of 2 with Me_3SiCl

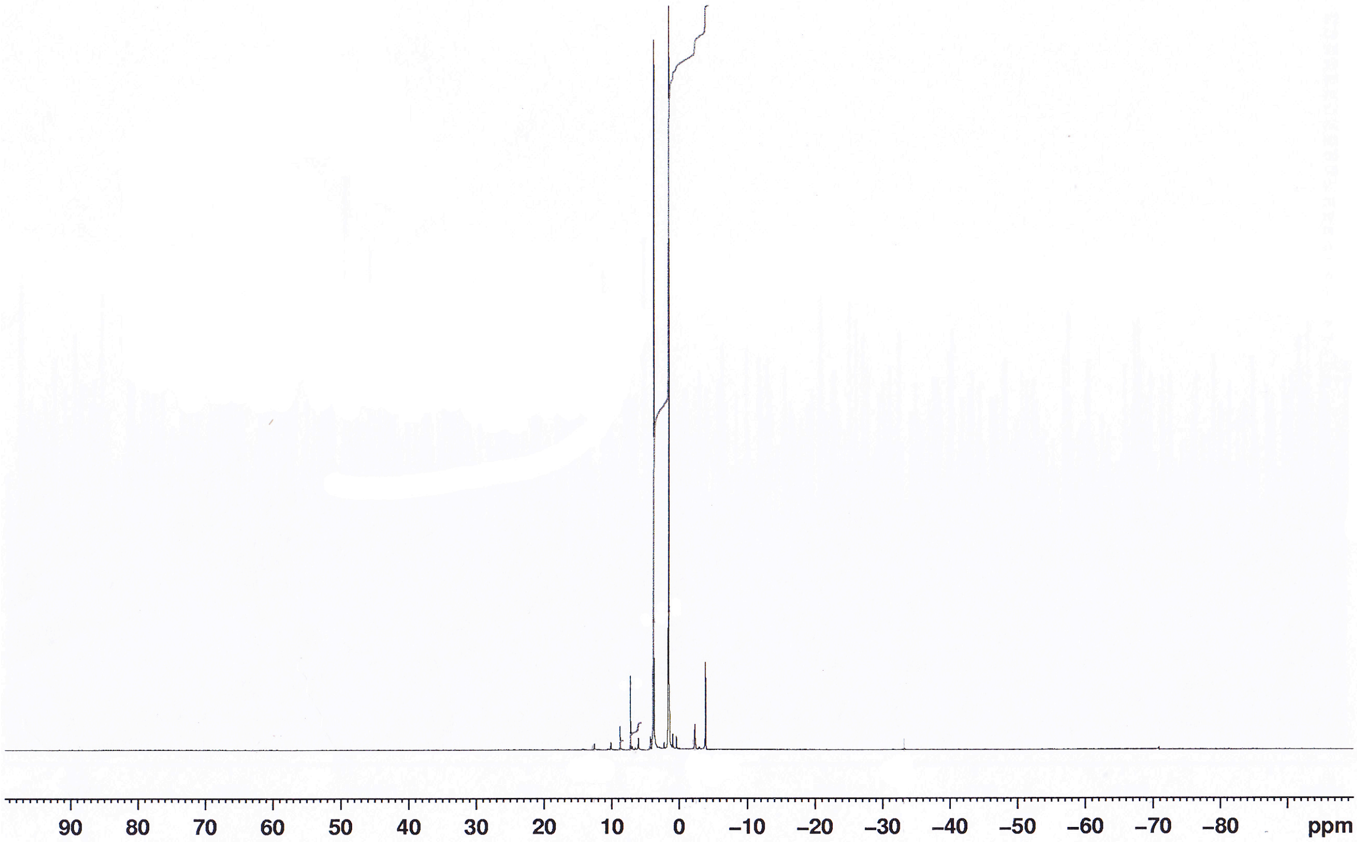

^{1}H NMR Spectrum of Reaction of 2 with LiCl

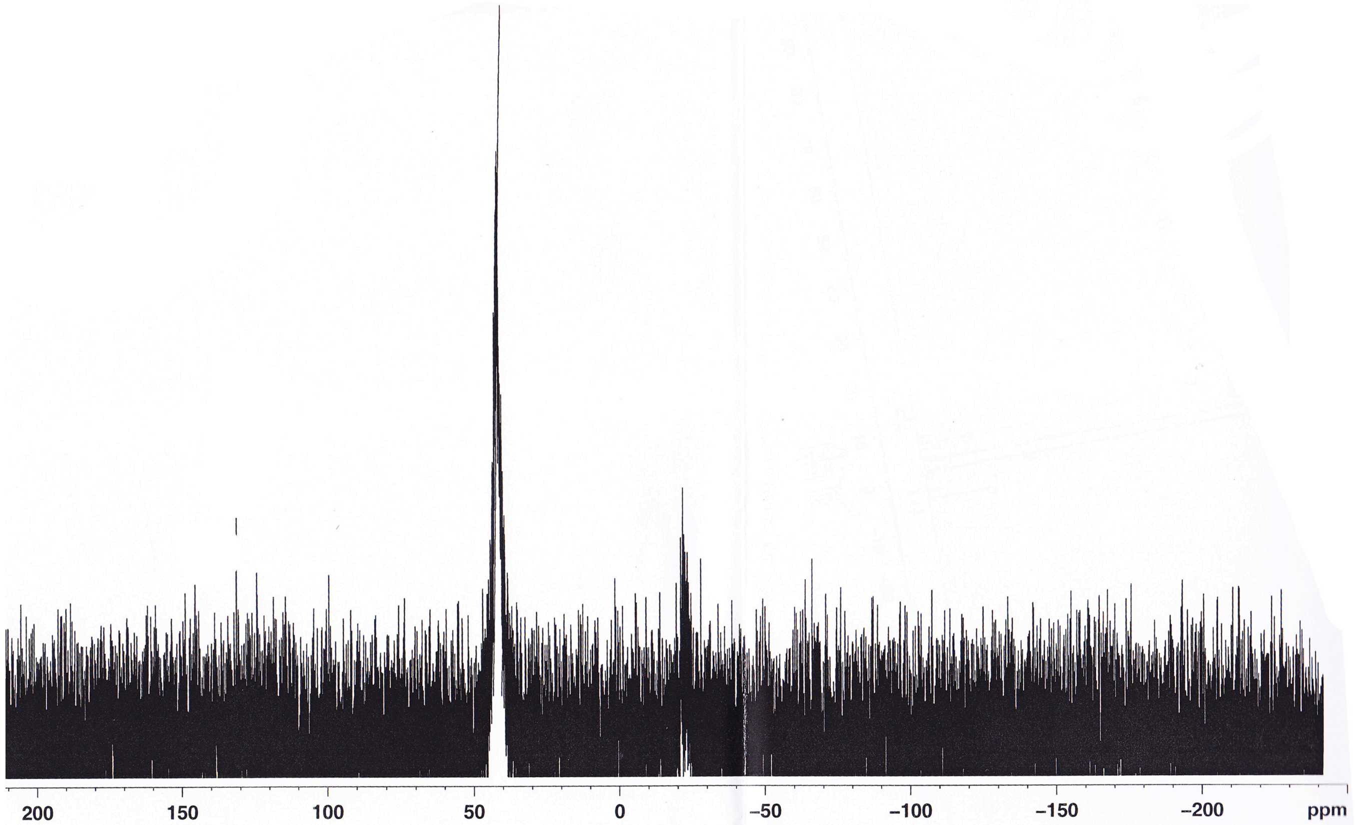

^{31}P NMR Spectrum of Reaction of 2 with LiCl

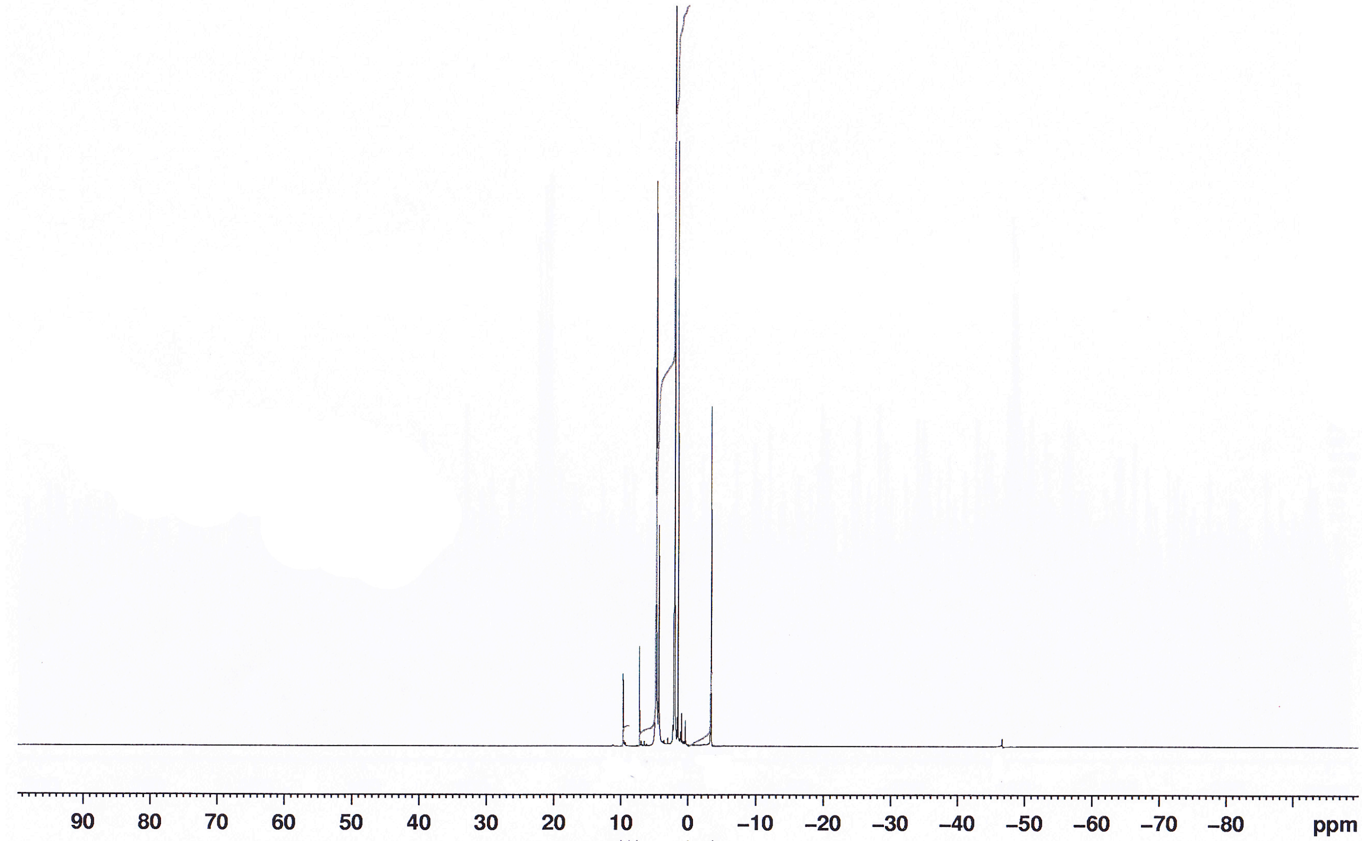

^{1}H NMR Spectrum of Reaction of 2 with MeI

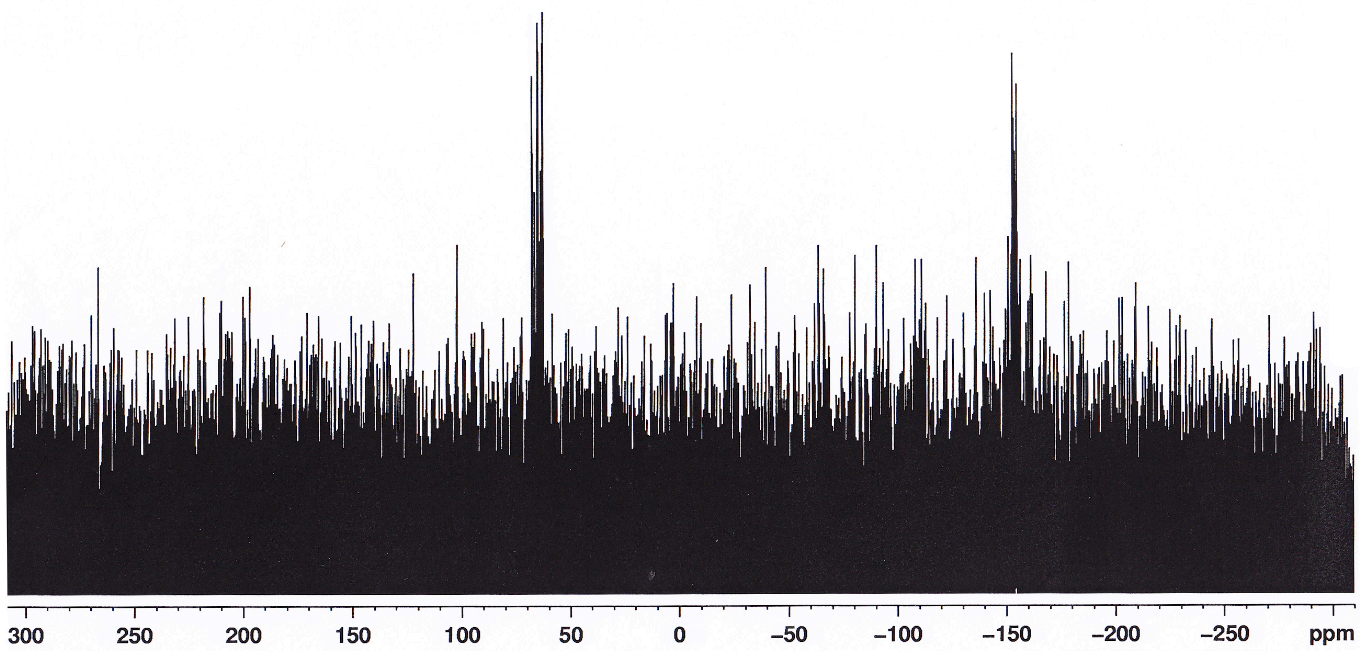

^{31}P NMR Spectrum of Reaction of 2 with MeI

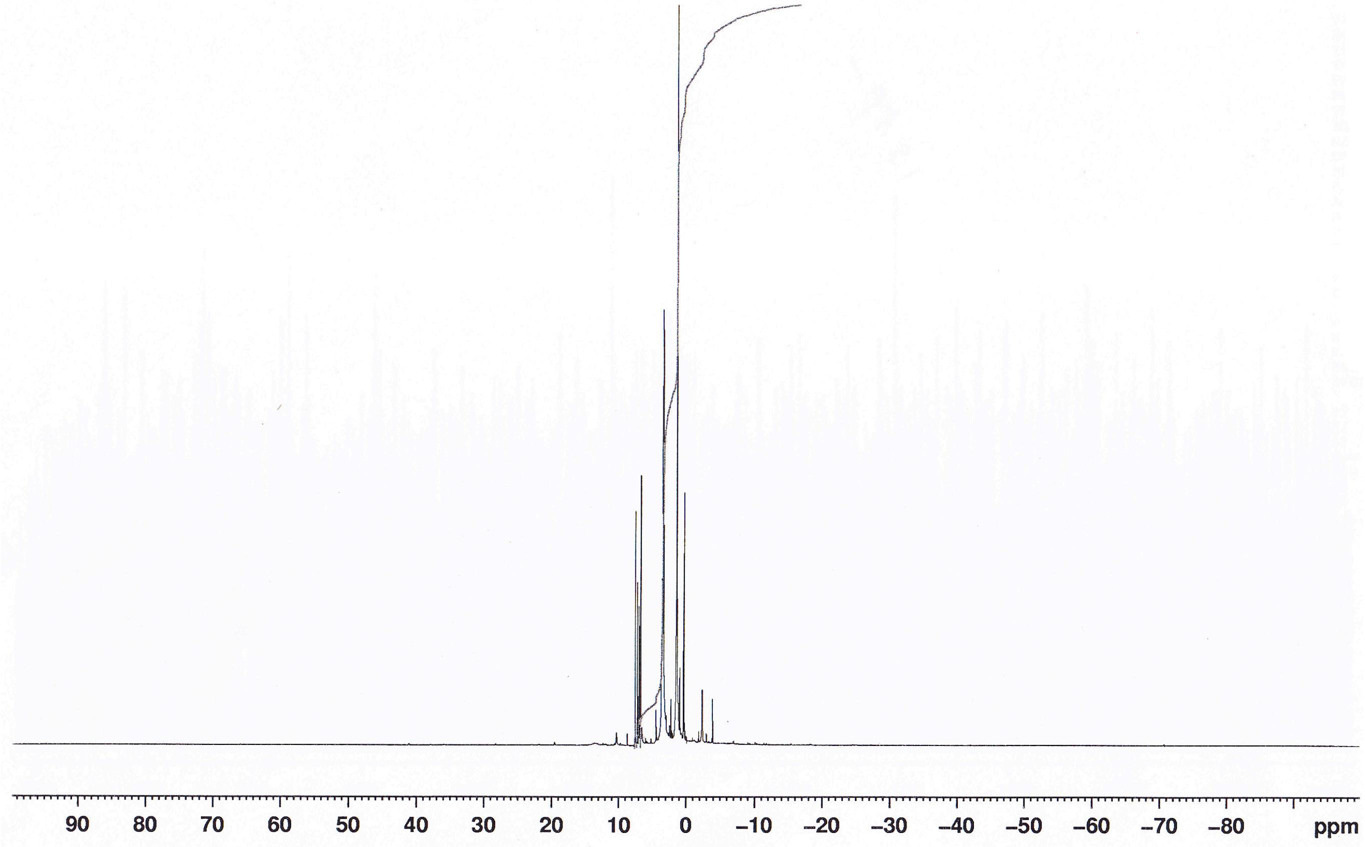

^{1}H NMR Spectrum of Reaction of 2 with PhI

The ^{31}P NMR spectrum of the reaction of **2** with PhI did not contain any obvious resonances.

DFT Calculations

General

Unrestricted geometry optimisations were attempted for the full model of **2** using coordinates derived from the X-ray crystal structure. However, the calculations proved to be intractable due to the size of the computational problem. Therefore, a single point energy calculation was carried out on the experimentally derived coordinates. The calculations were performed using the Amsterdam Density Functional (ADF) suite version 2012.01,[8,9] with Slater type orbital (STO) triple-ζ-plus polarization all-electron basis sets (from the ZORA/TZP database of the ADF suite). Scalar relativistic approaches were used within the ZORA Hamiltonian for the inclusion of relativistic effects and the local density approximation (LDA) with the correlation potential due to Vosko et al[10] was used in all of the calculations. Gradient corrections were performed using the functionals of Becke[11] and Perdew.[12]

Single Point Energy of **2**: -1332.08055725 eV

References

1. D. Patel, F. Moro, J. McMaster, W. Lewis, A. J. Blake, S. T. Liddle, *Angew. Chem. Int. Ed.* **2011**, *50*, 10388.
2. D. Patel, F. Tuna, E. J. L. McInnes, J. McMaster, W. Lewis, A. J. Blake, S. T. Liddle, *Dalton Trans.* **2013**, *42*, 5224.
3. W. Hönle, H. G. von Schnering, *Z. Anorg. Allg. Chem*. **1978**, *440*, 171.
4. G. Fritz and J. Härer, *Z. Anorg. Allg. Chem*. **1983**, *500*, 14.
5. W. Hönle, H. G. Von Schnering, A. Schmidpeter, G. Burget, *Angew. Chem. Int. Ed.* **1984**, *23*, 817.
6. M. Baudler, W. Faber, J. Hahn, *Z. Anorg. Allg. Chem.* **1980,** *469*, 15.
7. W. Hölderich, G. Fritz, *Z. Anorg. Allg. Chem.* **1979,** *457*, 127.
8. C. Fonseca Guerra, J. G. Snijders, G. te Velde E. J. Baerends, E. J. *Theor. Chem. Acc.* **1998**, *99*, 391.
9. G. te Velde, F. M. Bickelhaupt, S. J. A. van Gisbergen, C. Fonseca Guerra, E. J. Baerends, J. G. Snijders T. Ziegler, *J. Comput. Chem.* **2001**, *22*, 931.
10. S. H. Vosko, L. Wilk, M. Nusair, *Can. J. Phys.* **1980**, *58*, 1200.
11. A. D. Becke, *Phys. Rev. A.* **1988**, *38*, 3098.
12. J. P. Perdew, *Phys. Rev. B.* **1986**, *33*, 8822.
